# A Three-Month Consumption of Eggs Enriched with ω-3, ω-5 and ω-7 Polyunsaturated Fatty Acids Significantly Decreases the Waist Circumference of Subjects at Risk of Developing Metabolic Syndrome: A Double-Blind Randomized Controlled Trial

**DOI:** 10.3390/nu13020663

**Published:** 2021-02-18

**Authors:** Monique T. Ngo Njembe, Barbara Pachikian, Irina Lobysheva, Nancy Van Overstraeten, Louis Dejonghe, Eleonore Verstraelen, Marine Buchet, Catherine Rasse, Cécile Gardin, Eric Mignolet, Jean-Luc Balligand, Yvan Larondelle

**Affiliations:** 1 Louvain Institute of Biomolecular Science and Technology, UCLouvain, 1348 Louvain-la-Neuve, Belgium; monique.ngonjembe@uclouvain.be (M.T.N.N.); louis.dejonghe@uclouvain.be (L.D.); eleonore.verstraelen@uclouvain.be (E.V.); marine.buchet@uclouvain.be (M.B.); cecile.gardin@uclouvain.be (C.G.); eric.mignolet@uclouvain.be (E.M.); 2 Center of Investigation in Clinical Nutrition, UCLouvain, 1348 Louvain-la-Neuve, Belgium; barbara.pachikian@uclouvain.be; 3 Institute of Experimental and Clinical Research, Pole of Pharmacology and Therapeutics, UCLouvain, 1200 Woluwe-Saint-Lambert, Belgium; irina.lobysheva@uclouvain.be (I.L.); nancy.vanoverstraeten@uclouvain.be (N.V.O.); jl.balligand@uclouvain.be (J.-L.B.); 4 Louvain Institute of Data Analysis and Modeling in economics and statistics, Statistical Methodology and Computing Service, UCLouvain, 1348 Louvain-la-Neuve, Belgium; catherine.rasse@uclouvain.be

**Keywords:** Alpha-linolenic acid, docosahexaenoic acid, rumenic acid, punicic acid, enriched eggs, metabolic syndrome, waist circumference, obesity

## Abstract

Alpha-linolenic acid (ALA), docosahexaenoic acid (DHA), rumenic acid (RmA), and punicic acid (PunA) are claimed to influence several physiological functions including insulin sensitivity, lipid metabolism and inflammatory processes. In this double-blind randomized controlled trial, we investigated the combined effect of ALA, DHA, RmA and PunA on subjects at risk of developing metabolic syndrome. Twenty-four women and men were randomly assigned to two groups. Each day, they consumed two eggs enriched with oleic acid (control group) or enriched with ALA, DHA, RmA, and PunA (test group) for 3 months. The waist circumference decreased significantly (−3.17 cm; *p* < 0.001) in the test group. There were no major changes in plasma insulin and blood glucose in the two groups. The dietary treatments had no significant effect on endothelial function as measured by peripheral arterial tonometry, although erythrocyte nitrosylated hemoglobin concentrations tended to decrease. The high consumption of eggs induced significant elevations in plasma low-density lipoprotein (LDL)- and high-density lipoprotein (HDL)-cholesterol (*p* < 0.001), which did not result in any change in the LDL/HDL ratio in both groups. These results indicate that consumption of eggs enriched with ALA, DHA, RmA and PunA resulted in favorable changes in abdominal obesity without affecting other factors of the metabolic syndrome.

## 1. Introduction

Metabolic syndrome (MetS) encompasses a complex set of interrelated physiological and biochemical disorders, including disruption of lipid and glucose metabolism associated with vascular abnormalities and a pro-inflammatory state. Its most prominent components are abdominal obesity, hypertension, hyperglycaemia, hypertriglyceridemia and low level of high-density lipoprotein (HDL)-cholesterol [1]. These metabolic disorders dramatically increase the risk of type 2 diabetes, coronary diseases, and stroke. The International Diabetes Federation indicates that people with MetS have a fivefold greater risk of developing type 2 diabetes, and that they are three times more likely to have a heart attack or stroke and twice as likely to die from it [2]. The prevalence of MetS is estimated at around one quarter of the world’s population. Although this estimate varies depending on the age, ethnicity and gender of the population studied, the metabolic alterations are aggravated by lifestyle, including inactivity and dietary factors, mainly fats and some types of carbohydrates, which are strongly suspected of inducing both their development and complications [3,4].

There has been an increased interest over the past decade in understanding the metabolism of dietary lipids and their role in health. The special attention in fats has also come from the recognition that certain fatty acids are key regulators of gene expression and metabolic processes. In the worrisome context of the escalation of cardiovascular diseases (CVD) and type 2 diabetes, many researchers have studied the impact of some fatty acids on improving MetS factors.

Several follow-up studies reported that intakes of omega-3 polyunsaturated fatty acids (n-3 PUFA), including α-linolenic acid (ALA, C18:3c9,c12,c15) and its long-chain derivative, docosahexaenoic acid (DHA, C22:6c4,c7,c10,c13,c16,c19), are strongly associated with a lower risk of MetS [5,6]. Beneficial effects on the action of insulin have also been revealed by an inverse association found between the content of n-3 PUFA in the blood and insulin resistance [7,8]. A 3-month study in obese men indicated that supplementation with rumenic acid (RmA, C18:2c9,t11), an omega-7 conjugated linoleic acid (CLA) slightly decreased insulin sensitivity without altering serum lipids, glycaemia, body mass index (BMI) and body fat [9]. In contrast, Schmitt et al. [10] reported a preponderant effect of RmA in reducing insulinemia and counteracting insulin resistance after 60 days in obese and diabetic patients receiving a diet based on products rich in ALA and DHA. This suggests that combining RmA with n-3 PUFA would lead to beneficial effects on glycaemic parameters. Furthermore, punicic acid (PunA, C18:3c9,t11,c13), an omega-5 conjugated linolenic acid (CLnA), given to mice with diet-induced obesity, prevented excess body fat and improved insulin sensitivity [11]. Up to now, the combined effects of PunA, n-3 PUFA and RmA on obesity, serum lipids and glucose metabolism have not been established in both animals and humans.

In previous work, we have developed a method to enrich eggs with ALA, DHA, RmA and PunA through hen feeding [12]. The present study aimed at determining the effect of regular consumption of these eggs in adults at risk of MetS. Several key factors of the MetS were assessed, including glycosylated hemoglobin (HbA1c), insulin and fasting glucose parameters, blood pressure, abdominal obesity, lipids, and lipoproteins. We extended our study to the analysis of vascular health indicators. These include measurements of erythrocyte nitrosylated hemoglobin and endothelial function.

## 2. Materials and Methods

### 2.1. Study Population

Participants were recruited from the population aged 35 to 75, with a waist circumference greater than 80 cm for women and 94 cm for men, and practicing fewer than two hours of physical activity per week. Eligible participants completed a telephone screening to attend a medical check-up scheduled two weeks prior to the start of the study, at the Center of Investigation in Clinical Nutrition (Louvain-la-Neuve, Belgium). Comorbidity assessment and medical history review were performed for all subjects who provided information on allergies, food intolerances, smoking, drug addiction and current medication use.

Exclusion criteria were: uncontrolled hypertension (> 160/100 mmHg), type 1 or type 2 diabetes (fasting blood sugar ≥ 126 mg/dL or HbA1c ≥ 6.5%), CVD or high risk of CVD (total cholesterol > 239 mg/dL, low-density lipoprotein (LDL)-cholesterol > 159 mg/dL, HDL-cholesterol < 40 mg/dL, triglycerides > 200 mg/dL or familial history of premature cardiovascular incident); be involved in an active weight loss program or have experienced weight loss of more than 5 kg in the past three months, receiving medication or have other health issues that might compromise compliance with the study interventions. Pregnant, lactating and perimenopausal women with symptoms, as well as postmenopausal women for less than six months were also excluded from participating. All selected participants provided written informed consent. The study was conducted in accordance with the Declaration of Helsinki, and the Ethical Committee of the Cliniques universitaires Saint-Luc (Brussels, Belgium) approved the protocol. The trial is registered in the ClinicalTrials.gov database as NCT04583657.

### 2.2. Study Design

Participants were randomly assigned in a 1:1 ratio to either a test group or a control group. Randomization was stratified by gender using a computer-generated list of random numbers prepared by an independent statistician. In double-blind design, the test group received daily two eggs enriched with ALA, DHA, RmA and PunA, while the control group participants were given two eggs enriched with oleic acid (OA). The eggs were meant to be eaten cooked, preferably at breakfast or lunch, for three consecutive months. All eggs were produced at the University Farm of UCLouvain (Corroy-le-Grand, Belgium). The fatty acid profile of the eggs (Table 1) was checked weekly as described previously [12], to ensure it was constant throughout the study. An intervention staff strictly maintained blinding by packing the eggs in similar cardboard boxes on which a unique 3-letter code assigned to each participant was printed. Neither the assessors nor the participant knew which treatment the participant received. Boxes containing 16 eggs (2 additional eggs to prevent possible loss) were delivered to participants weekly. Upon delivery, uneaten eggs from the previous week were collected and participants responded to a questionnaire asking the occurrence, nature, severity and duration of potential adverse events, their link with the study, and whether participants had changed medication or taken a particular drug.

The three-month trial period included four medical visits: one at the start of the study (month 0 or baseline), when the subjects received the eggs for the first time, and three monthly visits (months 1, 2 and 3), the last of which ended the study. The primary endpoint chosen for the design of the study was the mean change in HbA1c from baseline to month 3. Other efficacy endpoints included obesity, serum lipids, insulin sensitivity, inflammation, and endothelial function. All assessments, except endothelial function, were performed at each monthly visit. The participants were requested not to change their habits regarding physical activity, to eat fish no more than twice a week, and to abstain from dietary supplements of n-3 PUFA, CLA or CLnA during the trial commitment period. The study was conducted from June 2019 to March 2020.

### 2.3. Dietary Assessment

Prior to medical visit, the subjects performed a 3-day dietary record. Standard food servings and a food atlas [13] were used to quantify household measures. The macronutrient composition of the diets was assessed using the French Agency for Food, Environmental and Occupational Health Safety database (ANSES-CIQUAL).

### 2.4. Anthropometric Measurements

Body weight was determined to the nearest 0.1 kg, height, waist, and hip circumferences to the nearest 0.1 cm, in subjects wearing light indoor clothing and without shoes. The waist-to-hip ratio (WHR) was calculated from these measurements. BMI was calculated by dividing the body weight in kilograms by the square of the height in meters. Body fat and lean body mass were calculated using a Tanita SC-240 bioelectric impedance analyzer (Tanita Europe BV, Amsterdam, The Netherlands) and manufacturer’s programmed equations.

### 2.5. Clinical Investigations

Blood samples were obtained from subjects after a 10- to 12-h overnight fast. Triglycerides, HDL-, non-HDL- and LDL-cholesterol, glycaemia, insulinemia, homeostasis model assessment for insulin resistance (HOMA-IR), quantitative insulin sensitivity check index (QUICKI), HbA1c, hemoglobin, hematocrit, erythrocytes, leucocytes, thrombocytes, high-sensitivity C-reactive protein (hs-CRP), aspartate aminotransferase (AST), alanine aminotransferase (ALT), gamma-glutamyltranspeptidase (GGT), urea, creatinine, estimated glomerular filtration rate (eGFR) and albumin were analyzed at the Clinique St Pierre (Ottignies, Belgium).

IL-6 and TNF-α assays were performed using Human IL-6 and TNF-α Quantikine HS ELISA kits (R&D Systems, Langley, UK) respectively, and oxidized LDL (ox-LDL) were measured employing a Mercodia oxidized LDL ELISA kit (Mercodia, Huissen, The Netherlands), according to manufacturer’s instructions.

### 2.6. Measurement of Nitrosylated Hemoglobin and Peripheral Artery Tonometry

On two occasions, at the first (month 0) and the last visit (month 3), the endothelial function was assessed by peripheral arterial tonometry and nitrosylated hemoglobin measurement.

Blood was drawn by venopuncture from the median cubital vein into a vacutainer tube containing EDTA (K2E, Vacutainer, BD-Plymouth, UK). A mixture of antioxidant solution (sodium ascorbate and N-acetylcysteine; final concentration, 5 mmol/L of both) was added into closed vacutainer using a Micro-Fine™ syringe prior the centrifugation to support the blood redox condition. The erythrocytes were collected after centrifugation (10 min, 800× *g*, at room temperature) from the bottom of the vacutainer tube into a 1 mL syringe and stored immediately at −80 °C. The concentration of heme-(Fe II)-nitrosyl-hemoglobin (HbNO) was assessed in the erythrocyte samples using the low-temperature Electron Paramagnetic Spectroscopy (EPR). The EPR spectra were recorded by an X-band EPR spectrometer (EMX-micro) (Bruker Instruments Inc., Billerica, MA, USA) with the following settings: microwave frequency, ~ 9.35 GHz; modulation frequency, 100 kHz; microwave power, 20 mW; modulation amplitude, 0.7 mT at 77 K using an EPR quartzfinger Dewar filled with liquid nitrogen. The erythrocyte concentration of the HbNO complex (5-coordinate α-heme-nitrosyl) was quantified from the intensity of the hyperfine components of the HbNO EPR signal (g-factor 2.01, Ahf = 16.8 G) after subtraction of the overlapping EPR signal of protein free radicals from the integral EPR spectrum of frozen erythrocytes following the method developed previously [14].

Endothelial vasodilation function was determined using the Endo-PAT 2000 device (Itamar Medical, Caesarea, Israel). Subjects were directed to rest in a quiet, dimly lit, temperature-controlled exam room. Two high-sensitive pneumatic probes (EndoPAT™, Itamar) were placed on the index fingers of the left and right hands, and plethysmographic signals in the index fingers were recorded throughout the test. A baseline record was performed for five minutes. For the 5-min occlusion phase, the blood pressure cuff placed on the right forearm was inflated to a supra-systolic pressure of 60 mmHg above the patient’s systolic pressure or to 200 mmHg, depending on the greater value between the two. Complete cessation of blood flow to the hand was verified by the absence of a peripheral arterial tone (PAT) signal from the occluded arm. Then the cuff was abruptly deflated as quickly as possible to initiate the post-occlusion recording phase (5 min). The reactive hyperemia index (RHI) was calculated by the device as the ratio of the post-to-pre-occlusion PAT signal in the ischemic arm (right arm), relative to the same ratio in the control arm (left arm), and corrected for baseline vascular tone [15]. Arterial stiffness was assessed by the augmentation index (AI), which was determined from the baseline resting pulse wave.

### 2.7. Statistics and Data Analysis

The study was designed to show a statistically significant 10% decrease in the primary endpoint (HbA1c) in the test group assuming a standard deviation of 0.8, using a two-sample t-test with 80% power and a 5% level of significance. PASS 14.0.7 software (NCSS, Kaysville, UT, USA) was used for the calculation based on 40 subjects per group and a dropout rate of 10%. Because of the lockdown measures intended to limit the spread of cases of covid-19 contamination from March 2020 on, the trial had to be stopped after 24 subjects had completed the experimental period.

Statistical analyses were performed using the software systems SAS 9.4 (SAS Institute Inc., Cary, NC, USA) and JMP Pro 15 SAS. Changes between groups (control and test) per visit (1, 2, 3 and 4) and between visits 1 and 4 for each group were analyzed using a linear mixed model for repeated measurements with subjects as random variable, and groups, visits, and their interaction as fixed independent variables. When the interaction was significant (*p*-value < 5%), pairwise comparisons were computed using t-tests followed by Bonferroni correction on selected combinations of groups and visits. When there was no significant interaction but the difference between groups or visits was significant, pairwise comparisons using Tukey’s test were computed. If necessary, a logarithm to the base 10 was used to fulfill the assumptions of the mixed model. Variables that were not normally distributed after the logarithmic transformation were analyzed using non-parametric methods. For analyses of within-group differences, the Wilcoxon matched-pairs signed rank test was used. The Wilcoxon Mann-Whitney two-sample test was used for analyses of differences between groups.

## 3. Results

### 3.1. Dietary Analysis and Tolerance

**Figure 1:**
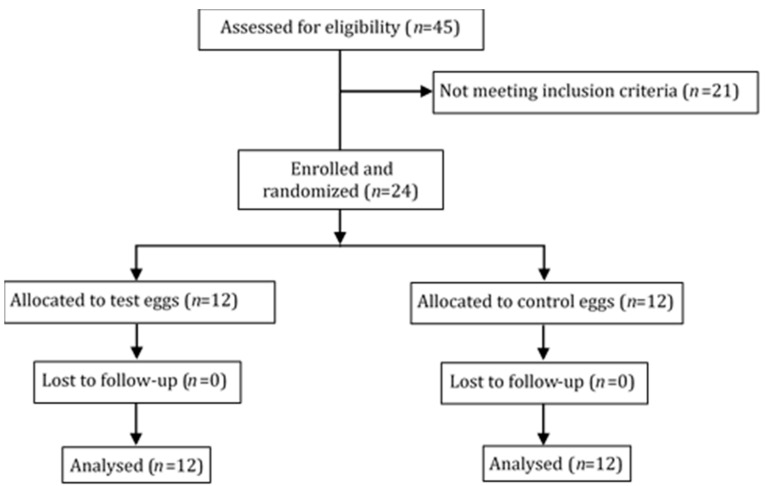
Recruitment flow diagram.

Twenty-four subjects, 12 in each treatment group, were included in the study as shown in Figure 1 and Table 2. All participants completed the trial. Adherence to study was assessed based on the number of uneaten eggs and the dietary record provided by the participants. The estimate of eggs consumed over those prescribed was greater than 95% in all participants.

Dietary analysis based on four 3-day food records, one completed before and the others completed during the study, showed that participants did not change their dietary intake with regard to energy, protein, carbohydrates, and fat (Table 3). Besides, the average daily intakes of energy and macronutrients did not differ significantly between groups. DHA intake increased in the test group. However, the difference in the DHA content of the control and the test eggs did not allow reaching a significant difference in the average daily intake of this n-3 PUFA between the two groups. In contrast, the consumption of approximately 1190 mg/day of RmA and 643 mg/day of PunA through the test eggs allowed a significant increase (*p* < 0.001) of their intakes by the test group. Subjects in both groups doubled their cholesterol intake during the study.

Participants experienced no adverse events except that two subjects in the test group reported mild nausea at some occasions. The following clinical chemistry variables: AST, ALT, GGT, hemoglobin, hematocrit, erythrocytes, leucocytes, platelets, albumin, total serum proteins, urea, creatinine and eGFR were relatively constant and remained within normal ranges from the beginning to the end of the study in both groups (data not shown).

### 3.2. Vital Signs and Anthropometrics

**Figure 2:**
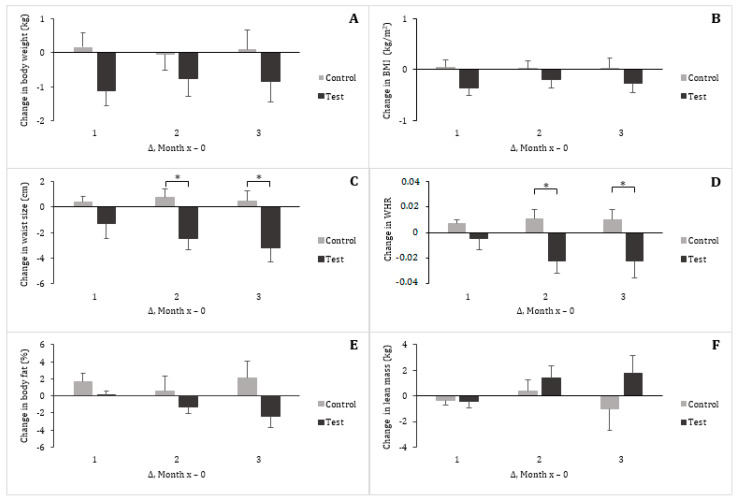
Changes (∆) in body weight (**A**), body mass index (BMI) (**B**), waist circumference (**C**), waist-to-hip ratio (WHR) (**D**), percentage of body fat (**E**) and lean body mass (**F**) in men and women daily consuming two eggs enriched in oleic acid (control group) or two eggs enriched in α-linolenic acid (ALA), docosahexaenoic acid (DHA), rumenic acid (RmA) and punicic acid (PunA) (test group), from the start of the study (Month 0) to months 1, 2 and 3 (Months x). Mean (± SEM). (*) for significant differences between control group and test group at *p* < 0.05, analyzed using *t*-test.

Heart rate, and systolic and diastolic blood pressures remained relatively constant in the two groups, without differences between them (Table 4). Body weight, BMI, waist circumference, and body composition were comparable across the two groups at the baseline (Table 2). When changes in these variables were analyzed over the course of the study, they displayed opposite patterns between the control group and the test group (Figure 2). However, the differences were not statistically significant for body weight, BMI, body fat and lean mass. The changes in waist circumference and in WHR were significant (*p* < 0.05) between the control and the test groups after two months of treatment. No significant difference was found within the control group from month 0 (baseline) to month 3, while waist circumference was reduced on average by 3.17 cm in the test group (*p* < 0.001, Table 4). To further explore the waist circumference response to dietary treatments, subjects were grouped by gender (Control group: females = 9 and males = 3; Test group, females = 8 and males = 4). No gender differences could be observed in any of the groups when performing this secondary analysis. The women and men in the control group showed a mean ± SEM reduction of 0.67 ± 1.02 and 0.01 ± 1.00 cm, respectively, while the women and men in the test group experienced a reduction in their waist circumference of 3.12 ± 1.41 and 3.25 ± 2.14 cm, respectively.

### 3.3. Insulin and Glucose Metabolism

**Figure 3:**
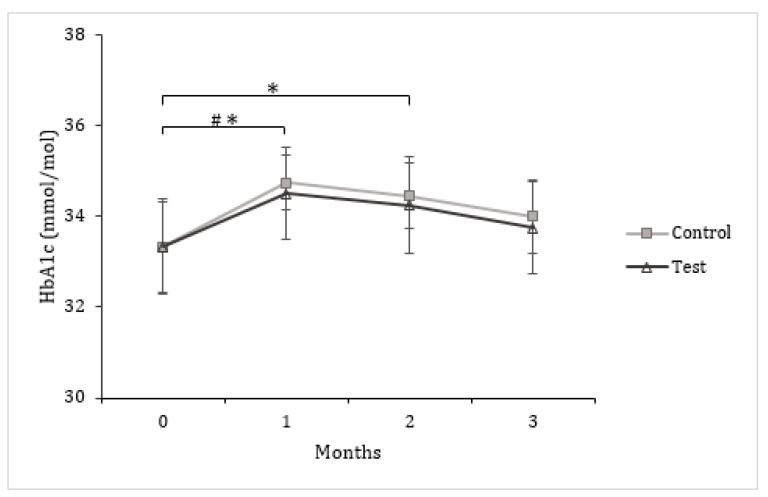
Glycosylated hemoglobin (HbA1c) in response to the daily consumption of two eggs enriched in oleic acid (OA) by the control group (⧠) or two eggs enriched in α-linolenic acid (ALA), docosahexaenoic acid (DHA), rumenic acid (RmA) and punicic acid (PunA) by the test group (∆), for 3 months. Mean (± SEM). Variables were logarithmically transformed prior to paired t-test, *p* < 0.05. (#) Significant difference compared to month 0 within the control group; (*) Significant difference compared to month 0 within the test group.

Blood glucose and insulin levels did not differ in the two groups at month 3 compared to month 0. HOMA-IR and QUICKI were also not affected by the different treatments (Table 4). HbA1c levels increased significantly from month 0 to month 1 (*p* < 0.01) in the control group and from month 0 to months 1 and 2 (*p* < 0.01 and *p* < 0.05, respectively) in the test group, and then returned at month 3 to levels similar to those at month 0 (Figure 3). There was no significant difference between the two groups regarding values for the same month.

### 3.4. Serum Triglycerides and Lipoproteins

**Figure 4:**
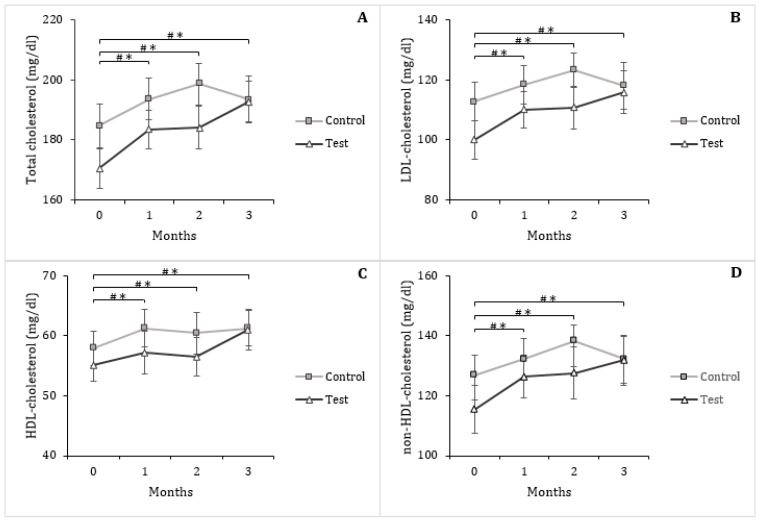
Total cholesterol (**A**), low-density lipoprotein (LDL)-cholesterol (**B**), high-density lipoprotein (HDL)-cholesterol (**C**) and non-HDL-cholesterol (**D**) in response to the daily consumption of two eggs enriched in oleic acid (OA) by the control group (⧠) or two eggs enriched in α-linolenic acid (ALA), docosahexaenoic acid (DHA), rumenic acid (RmA) and punicic acid (PunA) by the test group (∆) during 3 months. Mean (± SEM). Paired t-test, *p* < 0.001. (#) Significant difference compared to month 0 within the control group; (*) Significant difference compared to month 0 within the test group.

Changes in serum triglycerides were very small throughout the study and did not differ among treatment groups (Table 4). Total cholesterol concentrations were significantly increased in the two groups (*p* < 0.001), due to a significant increase in HDL and non-HDL-cholesterol (*p* < 0.001 in both groups) (Table 4, Figure 4). No difference between the groups was found. Analysis of within-group profiles showed that seven subjects in the control group had normal total cholesterol levels (<190 mg/dL) at the start of the study. Five subjects remained within normal levels, while the other two had an increase in total cholesterol above normal level by the first month of treatment. One subject in the control group with a high level at month 0 had a decrease in total cholesterol to a normal level at month 3. Concerning the test group, ten subjects had normal total cholesterol levels at month 0. Four of them exceeded the normal limit (two from month 1 and two at month 3), whereas two other subjects experienced an increase in total cholesterol above normal at months 1 and 2, then a decrease to normal levels at month 3.

### 3.5. Inflammation and Oxidative Stress

At month 0, there were no significant differences in plasma inflammatory markers between the groups, except that hs-CRP was significantly lower (*p* < 0.05) in the test group (Table 5). Both treatments had no effect on IL-6 and TNF-α levels. There was a within-group trend toward an increase in hs-CRP (*p* < 0.08) in the test group, while no change was observed in the control group. Plasma ox-LDL levels increased in both groups at month 3 compared to month 0. However, the increase was statistically significant only in the test group (*p* < 0.05). There was no statistically significant difference between the groups at months 0 and 3.

### 3.6. Erythrocyte Level of Nitrosylated Hemoglobin and Vascular Endothelial Function

As shown in Table 5, erythrocyte HbNO concentrations decreased in both study groups at month 3 (−24.06 nmol/L in the control group and −26.56 nmol/L in the test group). However, these differences were not statistically significant. The reactive hyperaemia indexes (RHI and LnRHI) did not differ among groups and throughout the study. The AI measurement for arterial stiffness was not significantly altered by either condition tested.

## 4. Discussion

In this double-blind randomized controlled parallel-group trial, we examined the effect of combined supplementation of ALA, DHA, RmA, and PunA on MetS components in healthy women and men, but with a high propensity to develop a MetS. OA was chosen as the control for its benefits in the management of CVD [16]. Its positive effects include lowering LDL-cholesterol and increasing HDL-cholesterol [17], and it may also help in good control of hypertriglyceridemia [18]. Chicken egg was of particular interest as a dietary carrier for supplementation since it is relatively high in lipids and has great culinary versatility. The good compliance and the zero dropout rate observed during the study indicate that both control and test eggs were well tolerated by the participants.

One of the findings of this study is that the waist circumference decreased significantly in the subjects given the test eggs, while body weight, percent of body fat and lean mass were relatively unchanged (Figure 2). This suggests changes in body fat distribution and a positive effect of these eggs on abdominal obesity. In the placebo-controlled, randomized clinical trial by Defina et al. [19] where overweight and obese individuals received n-3 PUFA supplementation (3 g/day for 6 months), no difference in weight and body composition was observed compared to the placebo group. These results were confirmed by subsequent studies in subjects with CVD and type 2 diabetes [20,21]. In contrast, multiple studies showed convincing effects of CLA on reducing weight and body fat in overweight and obese humans [22,23,24]. The isomers of CLA that have been the most investigated so far are RmA and t10,c12-CLA (C18:2t10,c12). In a meta-analysis examining the effectiveness of CLA in reducing body fat, Whigham et al. [25] highlighted the low number of human studies conducted with a single isomer. The few existing interventions mostly agree that RmA does not have significant effect on body composition in humans [9,26]. In agreement with this, numerous animal and some human studies have shown that of the two tested isomers of CLA, t10,c12-CLA specifically is responsible for the anti-obesity effects [27,28,29]. The effects observed in the present study could not be attributed to t10,c12-CLA, as it was not identified in the test eggs. Similar to our results, a patented lipid-based formulation containing PunA induced a reduction of waist circumference of 1.05 cm without change in body weight, body fat and muscle mass. Unfortunately, no indication of the amount of PunA used was provided [30].

Koba et al. [31] showed that PunA administered to mice induced a reduction in adipose tissue weight accompanied by an increased in carnitine-palmitoyltransferase activity in the liver and brown adipose tissue. The authors stated that the anti-obesity effect of PunA could be in part due to the stimulation of β-oxidation of fatty acids [32]. Furthermore, Lai et al. [33] found in 3T3-L1 cells that PunA decreased adipogenesis and preadipocyte differentiation by down-regulating the levels of peroxisome proliferator-activated receptor gamma (PPARγ), CCAAT/enhancer binding protein (C/EBP)β and C/EBPδ, as well as fatty acid synthase, a key enzyme in lipogenesis.

Fasting blood sugar and insulin remained unchanged in both groups throughout the study, as did HOMA and QUICKI since these indices are derived from blood sugar and insulinemia values. The HbA1c value provides information about the average concentration of glucose in the blood over the 2 to 3 months preceding the test. Surprisingly, the levels of HbA1c increased after one month of treatment before returning to baseline values at the end of the study (Figure 3). The reason of this transient modulation has not been identified. Since blood sugar, insulin and HbA1c were globally not affected between the start and the end of the study, we can state that the combined supplementation of ALA, DHA, RmA and PunA through egg consumption for 3 months has neither diabetogenic nor diabeto-mitigating effects.

One whole egg provides on average 177.19 mg (control egg) or 182.26 mg (test egg) of cholesterol (Table 1). Consequently, both groups reported a significant elevation in dietary cholesterol during the study period. The high blood cholesterol levels observed in both groups might therefore be attributed to the eggs consumed. Contradictorily, Fuller et al. [34], in a 3-month study in overweight or obese people with prediabetes or type 2 diabetes, found no significant difference in the change in triglycerides, total, LDL- and HDL-cholesterol between people consuming a high-egg diet (≥12 eggs/week) compared with those consuming a low-egg diet (<2 eggs/week). The same research group subsequently showed that the high-egg diet over a 12-month period produced no adverse effects on cardiovascular risk factors including triglycerides, total, LDL- and HDL-cholesterol, inflammatory markers, oxidative stress and glycaemia measurements [35].

Herron et al. [36] classified healthy people on the basis of their response to prolonged consumption of high dietary cholesterol. Hypo-responders, who experienced an increase in total cholesterol of < 0.05 mmol/L for each additional 100 mg of dietary cholesterol consumed, were 62.5%, whereas 37.5% experienced an increase in total cholesterol ≥0.41 mmol/L for each additional 100 mg and were considered hyper-responders. In the present study, 50% of the cohort exhibited a hyper-response to dietary cholesterol. Studies [36,37,38] indicated that dietary cholesterol intake from eggs significantly increases both serum LDL- and HDL-cholesterol, resulting in only a marginal change in the LDL-/HDL-cholesterol ratio. This is consistent with our findings. Indeed, the LDL-/HDL-cholesterol ratio did not change during the 3-month study and remained ≤ 2.01.

Historically, elevated LDL-cholesterol has been associated with an increased risk of CVD. However, non-HDL-cholesterol appears to be a better predictor for atherosclerotic vascular events [39]. Non-HDL-cholesterol is the total amount of lipoproteins containing apolipoprotein B (apoB), including very low-density lipoproteins and their metabolic remnants, intermediate density lipoproteins and chylomicrons. These lipoproteins can participate in atherogenesis by entering and getting trapped in the intima of the arterial wall [40,41]. The Multinational Cardiovascular Risk Consortium recently published an analytical tool to predict the long-term risk of CVD based on non-HDL-cholesterol levels. The reference value (hazard ratio, HR = 1.0) associated with the lowest risk of cardiovascular disease was set at 2.6 mmol/L or 100 mg/dL non-HDL-cholesterol in women and men. For individuals with the highest non-HDL-cholesterol levels (≥220 mg/dL; HR= 1.9 in women and 2.3 in men), the CVD event rate over 30 years was predicted to be approximately three-to-four-times higher than in those with the lowest non-HDL-cholesterol (<100 mg/dL) [42]. According to this assessment model, the HR value between the start and the end of the present study was slightly increased (from 1.2 to 1.3) in both groups, suggesting that the increase in non-HDL-cholesterol seen during the study did not provide a worrisome additional risk on the incidence of CVD in the subjects.

Another indicator of CVD risk is ox-LDL, which are generally considered to be proatherogenic. Elevated ox-LDL levels are reported to be strongly positively linked to both atherosclerosis and inflammatory markers, including CRP, TNF-α and IL-6 [43,44]. In our study, the test group treated with ALA, DHA, RmA and PunA enriched eggs experienced a moderate but significant increase in plasma ox-LDL levels (from 51.89 to 63.06 U/L, *p* < 0.05). Currently, reference ranges for ox-LDL have not been established. Based on a prospective observational study of an apparently healthy and non-MetS population, a threshold of ox-LDL < 60 U/L has been defined for people at low risk of developing MetS. A range of 60 to 69 U/L would characterize people at relatively moderate risk, while a cut-off ≥ 70 U/L would indicate a high risk [45]. There was no change in inflammatory markers TNF-α and IL-6 in the test group, although a non-significant upward trend in hs-CRP was seen, suggesting the development of low-grade inflammation already observed in the control group since the start of the study.

In accordance with our results, previous studies have reported that relatively high levels of PUFA in the diet increased LDL particles susceptibility to lipid peroxidation [46,47,48]. The type and the amount of fat in the diet influence the fatty acid composition of lipoproteins and cell membranes, and may therefore affect the sensitivity of LDL and cells to oxidative damage. The more unsaturated the fatty acid, the more easily it oxidizes. Therefore, OA is less sensitive to oxidation compared with ALA, DHA, RmA and PunA [49]. If the latter are abundant in the LDL particles, they will promote the formation of ox-LDL. Yang et al. [50] comparing the oxidative stabilities of CLnA and CLA with their corresponding non-conjugated counterparts, ALA and linoleic acid, found that CLnA were the most unstable, followed by CLA, ALA and linoleic acid, in descending order. Unsurprisingly, DHA has also been found to enhance the susceptibility of cells to oxidative stress [46].

In addition, dietary fatty acids contribute to the pro-oxidant activity of arterial wall cells, since the fatty acid composition of the cell membrane influences cellular formation of reactive oxygen species (ROS) [48]. The increased production of ROS impairs nitric oxide (NO) bioavailability and prevents it from inducing vascular smooth muscle relaxation, which may lead to endothelial dysfunction. After a 3-month consumption of eggs enriched with mono- or poly-unsaturated fatty acids, erythrocyte HbNO levels were slightly reduced, indicating vascular oxidative stress and a reduction of NO bioavailability [51]. This is consistent with the weakly elevated hs-CRP (around 0.20 mg/dL) and the increase in ox-LDL seen in the two groups at month 3. High cholesterol levels have also been found to decrease the production of endothelial NO [52]. As the erythrocyte level of HbNO was shown to be a surrogate index of the endothelial NO production [53], this reduction of NO production was manifested by a decrease in HbNO levels, and was consistent with the observed cholesterol increase in both groups. However, the moderate decrease of the level of HbNO was not associated with impairment of the reactive hyperemia blood flow response assessed by peripheral arterial tonometry, likely due to the reduced number of subjects and the fact that peripheral arterial tonometry reflects more than an NO-dependent control.

## 5. Conclusions

This study, to our knowledge, is the first exploring the effects of eggs enriched with ALA, DHA, RmA and PunA on some metabolic parameters. The findings indicate that the consumption of these eggs during three months by subjects at high risk of MetS leads to a significant reduction in abdominal obesity, without improving other components of MetS including glycaemia-associated parameters. Although polyunsaturated fatty acids are expected to reduce the risk of CVD, they are prone to lipid peroxidation, and together with high cholesterol levels, may alter some markers of vascular oxidative stress. A limitation of this study is the small sample size, which may have prevented the emergence of certain differences between the two conditions of the study. Relevant outcomes (abdominal obesity, ox-LDL, HbNO), as well as their durability, deserve to be further studied on a larger sample and over a longer time frame. Another limitation is that to aid adherence, no strict dietary advice has been given regarding egg consumption. The participants did not change their eating behavior during the study period and the nutritional intake of cholesterol was not balanced. For future studies, prescribed diets or dietary guidance should be provided to participants in order to balance the cholesterol intake associated with egg consumption.

## Figures and Tables

**Table 1:** Composition of fatty acids, total lipid, and cholesterol content of eggs.

	Control	Test
Fatty acids (mg/egg)		
Lauric acid (C12:0)	3.93 ± 0.08	3.95 ± 0.08
Myristic acid (C14:0)	9.47 ± 0.21	12.04 ± 0.28
Palmitic acid (C16:0)	993.91 ± 17.29	1008.68 ± 16.67
Palmitoleic acid (C16:1c9)	56.24 ± 2.00	51.03 ± 1.81
Stearic acid (C18:0)	344.47 ± 5.90	386.41 ± 5.52
Oleic acid (C18:1c9)	2689.32 ± 33.29	1115.25 ± 25.97
Cis-vaccenic acid (C18:1c11)	70.96 ± 1.25	35.58 ± 1.04
Linoleic acid (C18:2c9,c12)	543.01 ± 10.58	622.70 ± 13.30
Gamma-linolenic acid (C18:3c6,c9,c12)	3.52 ± 0.13	4.20 ± 0.13
Alpha-linolenic acid (C18:3c9,c12,c15)	14.31 ± 0.34	105.19 ± 4.04
Rumenic acid (C18:2c9,t11)	2.06 ± 0.21	595.16 ± 10.64
Punicic acid (C18:3c9,t11,c13)	ND	321.59 ± 9.25
Dihomo-gamma-linolenic acid (C20:3c8,c11,c14)	6.50 ± 0.13	7.55 ± 0.17
Arachidonic acid (C20:4c5,c8,c11,c14)	97.89 ± 1.46	62.40 ± 1.45
Eicosapentaenoic acid (C20:5c5,c8,c11,c14,c17)	0.05 ± 0.02	2.58 ± 0.11
n-6 Docosapentaenoic acid (C22:5c4,c7,c10,c13,c16)	23.35 ± 0.70	4.47 ± 0.24
Docosahexaenoic acid (C22:6c4,c7,c10,c13,c16,c19)	37.72 ± 0.69	82.81 ± 1.89
Σ SFA	1378.63 ± 22.40	1445.70 ± 22.31
Σ MUFA	2833.56 ± 35.64	1221.60 ± 28.01
Σ n-6 PUFA	691.28 ± 119.13	716.03 ± 138.72
Σ n-3 PUFA	61.55 ± 1.02	230.16 ± 6.15
Total lipids (% by weight of fresh yolk)	31.08 ± 0.59	32.45 ± 0.44
Total cholesterol (mg/egg)	177.19 ± 5.40	182.26 ± 10.13

Values as “mean ± SEM”. ND: not detected; SFA: saturated fatty acids; MUFA: monounsaturated fatty acids; n-6 PUFA: omega-6 polyunsaturated fatty acids; n-3 PUFA: omega-3 polyunsaturated fatty acids.

**Table 2:** Baseline characteristics of participants in control and test groups.

	Control (*n* = 12)		Test (*n* = 12)		*p*-Value ^a^
	Mean ± SEM	Range	Mean ± SEM	Range	
Sex (women/men)	9/3	-	8/4	-	-
Age (years)	51.67 ± 2.39	42–71	45.58 ± 2.43	35–59	0.105
Body weight (kg)	76.23 ± 2.64	66.4–93.8	74.34 ± 4.18	59.1–98.1	0.402
BMI (kg/m^2^)	25.98 ± 0.75	21.9–30.3	26.04 ± 1.20	20.3–35.6	0.402
Waist circumference (cm)	93.38 ± 2.69	82–111	92.21 ± 2.79	83–111	0.840
Hip circumference (cm)	106.38 ± 1.49	99–115	102.79 ± 2.91	92–132	0.296
WHR	0.88 ± 0.03	0.8–1.0	0.89 ± 0.02	0.8–1.1	0.564
Body fat (%)	33.48 ± 1.71	22.5–42.3	31.83 ± 2.24	18.1–48	0.624
Lean body mass (kg)	50.13 ± 2.56	42.7–66.6	49.81 ± 2.65	37.4–64.9	1.000
Hemoglobin (g/dL)	13.89 ± 0.34	12.2–15.5	14.18 ± 0.49	12–16.71	0.751
Hematocrit (%)	41.6 ± 0.89	36.1–46.4	42.20 ± 1.24	36.2–48.2	0.773
Red blood cells (10^6^/mm^3^)	4.66 ± 0.10	4.08–5.22	4.75 ± 0.16	3.93–5.66	0.977
White blood cells (10^3^/mm^3^)	5.28 ± 0.27	3.95–7.13	5.52 ± 0.26	3.93–7.27	0.507
Platelets (10^3^/mm^3^)	226 ± 14.09	144–301	222.17 ± 15.07	144–301	0.665
AST (UI/L)	18.83 ± 1.07	14–27	21.64 ± 2.13	14–35	0.477
ALT (UI/L)	17.25 ± 2.07	10–34	20.67 ± 2.69	10–44	0.247
GGT (UI/L)	15.50 ± 0.97	10–20	18.17 ± 2.52	9–36	0.664
Urea (mg/dL)	30.75 ± 1.50	20–38	33 ± 2.17	23–50	0.401
Creatininemia (mg/dL)	0.79 ± 0.02	0.67–0.93	0.86 ± 0.04	0.67–1.09	0.247
eGFR (mL/min)	92.42 ± 2.28	82–104	90.75 ± 3.35	73–112	0.623
Albumin (g/dL)	4.30 ± 0.09	3.90–4.90	4.31 ± 0.06	3.90–4.60	0.640
Total protein (g/dL)	6.94 ± 0.14	6–7.50	6.78 ± 0.12	6.20–7.30	0.271

*n* = number of subjects. ^a^ Differences between the two groups were analyzed using Wilcoxon Mann-Whitney two-sample test (significant at *p*-value ˂ 0.05). BMI: body mass index; WHR: waist-to-hip ratio; AST: aspartate aminotransferase; ALT: alanine aminotransferase; GGT: gamma-glutamyl transpeptidase; eGFR: estimated glomerular filtration rate.

**Table 3:** Dietary intake of study participants based on the 3-day food record.

	Months			
	0	1	2	3
Energy (kcal/day)				
Control	1865.2 ± 159.06 ^a^	1811.92 ± 146.90 ^a^	1805.75 ± 145.23 ^a^	1818.33 ± 161.42 ^a^
Test	1782.45 ± 100.32 ^a^	1531.36 ± 81.53 ^a^	1637.36 ± 105.59 ^a^	1538.27 ± 137.10 ^a^
Protein (% of daily energy)				
Control	16.00 ± 0.93 ^a^	17.67 ± 1.24 ^a^	17.92 ± 1.12 ^a^	17.50 ± 0.72 ^a^
Test	15.18 ± 1.23 ^a^	17.27 ± 1.15 ^a^	15.91 ± 0.94 ^a^	18.55 ± 1.67 ^a^
Carbohydrates (% of daily energy)				
Control	43.50 ± 1.92 ^a^	38.67 ± 1.31 ^a^	40.75 ± 1.72 ^a^	40.33 ± 1.68 ^a^
Test	38.27 ± 2.29 ^a^	39.45 ± 2.18 ^a^	36.64 ± 2.13 ^a^	36.09 ± 2.02 ^a^
Fiber (g/day)				
Control	21.4 ± 1.54 ^a#^	20.00 ± 16.77 ^a#^	19.17 ± 15.67 ^a#^	20.58 ± 1.56 ^a#^
Test	16.45 ± 1.77 ^a#^	14.18 ± 1.32 ^a#^	14.09 ± 1.47 ^a#^	14.09 ± 135 ^a#^
Lipids (% of daily energy)				
Control	35.62 ± 2.49 ^a^	36.79 ± 1.74 ^a^	36.19 ± 1.71 ^a^	36.06 ± 1.73 ^a^
Test	38.08 ± 2.19 ^a^	36.58 ± 2.02 ^a^	40.90 ± 2.17 ^a^	40.09 ± 1.99 ^a^
SFA (% of daily energy)				
Control	16.44 ± 1.25 ^a^	14.80 ± 1.05 ^a^	14.63 ± 1.22 ^a^	14.25 ± 0.82 ^a^
Test	15.22 ± 0.88 ^a^	15.82 ± 1.42 ^a^	17.59 ± 1.15 ^a^	16.81 ± 0.83 ^a^
MUFA (% of daily energy)				
Control	10.29 ± 0.95 ^a^	12.70 ± 0.80 ^b^	12.27 ± 0.80 ^ab^	12.88 ± 0.74 ^b^
Test	13.43 ± 1.51 ^a^	11.40 ± 0.85 ^a^	11.86 ± 1.43 ^a^	11.09 ± 1.03 ^a^
PUFA (% of daily energy)				
Control	3.40 ± 0.36 ^a^	4.80 ± 0.45 ^b^	4.39 ± 0.31 ^ab^	4.58 ± 0.34 ^b^
Test	4.27 ± 0.62 ^a^	5.02 ± 0.31 ^a^	5.00 ± 0.44 ^a^	5.56 ± 0.54 ^a^
Alpha-linolenic acid (mg/day)				
Control	593.9 ± 132.63 ^a^	930.58 ± 136.68 ^a^	728.33 ± 103.98 ^a^	679.17 ± 129.75 ^a^
Test	697.81 ± 119.61 ^a^	675 ± 87.35 ^a^	629.73 ± 100.52 ^a^	737.82 ± 168.48 ^a^
Rumenic acid (mg/day)				
Control	125.9 ± 19.23 ^a^	116.08 ± 28.92 ^a##^	108.75 ± 19.82 ^a##^	117.58 ± 17.81 ^a##^
Test	92.64 ± 19.93 ^a^	1167.27 ± 116.94 ^b##^	1106.55 ± 131.88 ^b##^	1187.73 ± 112.41 ^b##^
Punicic acid (mg/day)				
Control	0.00 ± 0.00 ^a^	0.00 ± 0.00 ^a###^	0.00 ± 0.00 ^a###^	0.00 ± 0.00 ^a###^
Test	0.00 ± 0.00 ^a^	643.18 ± 18.51 ^b###^	643.18 ± 18.51 ^b###^	643.18 ± 18.51 ^b###^
Docosahexaenoic acid (mg/day)				
Control	148.20 ± 63.85 ^a^	216.17 ± 51.55 ^a^	230.42 ± 59.12 ^a^	265.50 ± 126.67 ^a^
Test	101.55 ± 37.62 ^a^	189.64 ± 14.02 ^ab^	278.27 ± 72.18 ^b^	232.73 ± 34.41 ^b^
Cholesterol (mg/day)				
Control	284.30 ± 38.36 ^a^	621.50 ± 34.93 ^b^	581.08 ± 19.91 ^b^	593.5 ± 17.38 ^b^
Test	262.45 ± 38.25 ^a^	571 ± 21.43 ^b^	589.09 ± 54.26 ^b^	573.45 ± 23.66 ^b^

Values as “mean ± SEM”. Differences between the values were determined using *t*-test. Values in the same row with no common superscripts (a, b) are significantly different, *p* ˂ 0.05. The difference between the two groups within the same month was significant at *p* ˂ 0.05 (#), *p* ˂ 0.001 (##) or *p* ˂ 0.0001(###). SFA: saturated fatty acids; MUFA: monounsaturated fatty acids; PUFA: polyunsaturated fatty acids.

**Table 4:** Changes in blood pressure, anthropometric measurements, glycaemic parameters and serum lipids between start and end of study.

		Control (*n* = 12)			Test (*n* = 12)	
	Month 0	Month 3	∆, Month 3-0	Month 0	Month 3	∆, Month 3-0
Resting heart rate (beats/min)	68.33 ± 2.23	66.50 ± 2.22	−1.83 ± 2.22	66.25 ± 2.51	69.50 ± 2.24	3.25 ± 2.45
Systolic blood pressure (mmHg)	128.33 ± 4.80	122.33 ± 3.17	−6.00 ± 3.01	122.33 ± 4.76	118.17 ± 5.59	−4.17 ± 4.39
Diastolic blood pressure (mmHg)	78.50 ± 3.27	76.25 ± 1.85	−2.25 ± 2.58	77.08 ± 3.44	73.33 ± 3.57	−3.75 ± 2.45
Weight (kg)	76.23 ± 2.64	76.32 ± 2.76	0.09 ± 0.58	74.34 ± 4.18	73.48 ± 4.01	−0.86 ± 0.58
BMI (kg/m^2^)	25.98 ± 0.75	26.01 ± 0.79	0.03 ± 0.19	26.04 ± 1.20	25.78 ± 1.16	−0.26 ± 0.19
Waist circumference (cm) ^a^	93.38 ± 2.69	93.88 ± 2.69	0.50 ± 0.79	92.21 ± 2.79	89.04 ± 2.60	−3.17 ± 1.12 **
Hip circumference (cm)	106.38 ± 1.49	105.63 ± 1.44	−0.75 ± 0.53	102.79 ± 2.91	101.83 ± 2.57	−0.96 ± 1.11
WHR	0.88 ± 0.03	0.89 ± 0.02	0.01 ± 0.01	0.90 ± 0.02	0.88 ± 0.03	−0.02 ± 0.01
Body fat (%)	33.48 ± 1.71	35.62 ± 2.30	2.14 ± 1.95	31.83 ± 2.24	29.48 ± 2.64	−2.35 ±1.37
Lean mass (kg)	50.13 ± 2.56	49.08 ± 2.43	−1.04 ± 1.64	49.81 ± 2.65	51.54 ± 3.00	1.73 ± 1.44
Fasting blood glucose (mg/dL) ^a^	86.75 ± 1.44	86.42 ± 2.90	−0.33 ± 2.43	87.25 ± 2.69	89.42 ± 2.42	2.17 ± 2.77
Fasting insulin (mUI/mL) ^a^	6.47 ± 0.66	5.76 ± 0.54	−0.71 ± 0.48	6.24 ± 0.82	7.38 ± 1.00	1.13 ± 0.90
HOMA-IR	1.40 ± 0.16	1.25 ± 0.14	−0.15 ± 0.12	1.38 ± 0.20	1.68 ± 0.27	0.30 ± 0.25
QUICKI	0.37 ± 0.03	0.38 ± 0.01	0.01 ± 0.004	0.38 ± 0.04	0.36 ± 0.01	−0.01 ± 0.01
HbA1c (mmol/mol) ^a^	33.33 ± 1.00	34.00 ± 0.82	0.67 ± 0.31	33.33 ± 1.05	33.75 ± 1.02	0.42 ± 0.29
Triglycerides (mg/dL)	70.17 ± 5.74	70.83 ± 6.80	0.67 ± 7.29	77.25 ± 9.98	79.17 ± 11.82	1.92 ± 9.33
Total cholesterol (mg/dL)	184.67 ± 7.21	193.50 ± 7.95	8.83 ± 4.44 **	170.50 ± 6.66	192.83 ± 6.67	22.33 ± 5.46 **
LDL-cholesterol (mg/dL)	112.67 ± 6.39	118.08 ± 7.88	5.42 ± 3.60 **	100.00 ± 6.29	116.00 ± 7.06	16.00 ± 4.51 **
HDL-cholesterol (mg/dL)	57.92 ± 2.87	61.25 ± 2.92	3.33 ± 1.29 **	55.08 ± 2.70	61.00 ± 3.36	5.92 ± 1.64 **
LDL/HDL	1.99 ± 0.14	1.97 ± 0.15	−0.03 ± 0.05	1.90 ± 0.19	2.01 ± 0.20	0.10 ± 0.07
non-HDL-cholesterol (mg/dL)	126.75 ± 6.66	132.25 ± 7.38	5.50 ± 3.73 **	115.42 ± 8.09	131.83 ± 8.28	16.42 ± 4.53 **

*n* = number of subjects. ∆: change between the two months. Values as “mean ± SEM”. BMI: body mass index; WHR: waist-to-hip ratio; HOMA-IR: homeostasis model assessment for insulin resistance; QUICKI: quantitative insulin sensitivity check index; HbA1c: glycosylated hemoglobin; LDL: low-density lipoprotein; HDL: high-density lipoprotein. ^a^ Variables were logarithmically transformed prior to paired t-test within groups. (**) Significant change from month 0 to 3, *p* ˂ 0.001.

**Table 5:** Changes in inflammation, oxidative stress and vascular health parameters between start and end of study.

		Control (*n* = 12)			Test (*n* = 12)	
	Month 0	Month 3	∆, Month 3-0	Month 0	Month 3	∆, Month 3-0
hs-CRP (mg/dL)	0.23 ± 0.06 ^#^	0.19 ± 0.04	−0.03 ± 0.06	0.09 ± 0.02^#^	0.21 ± 0.06	0.11 ± 0.06
IL-6 (pg/mL)	1.29 ± 0.21	1.29 ± 0.24	0.00 ± 0.27	1.20 ± 0.16	1.69 ± 0.36	0.49 ± 0.36
TNF-α (pg/mL)	0.87 ± 0.08	0.86 ± 0.07	−0.01 ± 0.04	0.79 ± 0.05	0.85 ± 0.05	0.06 ± 0.03
Ox-LDL (U/L)	55.66 ± 4.14	59.13 ± 3.75	3.47 ± 3.51	51.89 ± 4.29 *	63.06 ± 3.94 *	11.16 ± 9.81
HbNO (nmol/L)	102.50 ± 10.37	78.44 ± 13.59	−24.06 ± 17.48	110.42 ± 18.29	83.86 ± 16.39	−26.56 ± 14.33
RHI	2.30 ± 0.18	2.17 ± 0.17	−0.18 ± 0.20	2.32 ± 0.19	1.98 ± 0.19	−0.34 ± 0.12
LnRHI	0.81 ± 0.08	0.74 ± 0.09	−0.09 ± 0.11	0.81 ± 0.08	0.63 ± 0.10	−0.17 ± 0.06
AI (%)	18.74 ± 6.20	20.50 ± 8.12	1.76 ± 4.33	12.60 ± 6.33	14.94 ± 5.66	2.33 ± 2.66
AI@75bpm (%)	8.92 ± 6.21	12.95 ± 9.01	4.03 ± 5.39	4.64 ± 5.66	7.34 ± 5.22	2.69 ± 2.53

*n* = number of subjects. ∆: change between the two months. Values as “mean ± SEM”. (*) Significant difference within groups analyzed using Wilcoxon matched-paired signed rank test, *p* ˂ 0.05. (#) Significant difference in the same month between the two groups analyzed using Wilcoxon Mann-Whitney two-sample test, *p* ˂ 0.05. hs-CRP: high-sensitivity C-reactive protein; IL-6: interleukin 6; TNF-α: tumor necrosis factor alpha; Ox-LDL: oxidized low-density lipoprotein; HbNO: nitrosylated hemoglobin; RHI: reactive hyperaemia index; AI: augmentation index; AI@75bpm: augmentation index adjusted to a heart rate of 75 beats per minute.

## Data Availability

Data sharing is not applicable to this article.
